# Increasing the reach: Involving local Muslim religious teachers in a behavioral intervention to eliminate urogenital schistosomiasis in Zanzibar

**DOI:** 10.1016/j.actatropica.2016.08.004

**Published:** 2016-11

**Authors:** Mike Celone, Bobbie Person, Said M. Ali, Jameelat H. Lyimo, Ulfat A. Mohammed, Alippo N. Khamis, Yussra S. Mohammed, Khalfan A. Mohammed, David Rollinson, Stefanie Knopp

**Affiliations:** a Zanzibar Neglected Tropical Diseases Program, Ministry of Health, P.O. Box 236, Zanzibar Town, Unguja, United Republic of Tanzania; b Consultant of the Schistosomiasis Consortium for Operational Research and Evaluation, University of Georgia, Athens, GA, United States of America; c Public Health Laboratory – Ivo de Carneri, P.O. Box 122, Chake Chake, Pemba, United Republic of Tanzania; d Wolfson Wellcome Biomedical Laboratories, Department of Life Sciences, Natural History Museum, Cromwell Road, London SW7 5BD, United Kingdom; e Swiss Tropical and Public Health Institute, Socinstrasse 57, CH-4002 Basel, Switzerland; f University of Basel, Petersplatz 1, CH-4003 Basel, Switzerland

**Keywords:** EKBB, Ethikkommission beider Basel, HCD, Human Centered Design, HIV, Human Immunodeficiency Virus, MDA, mass drug administration, NCEZID, National Center for Emerging Zoonotic Diseases, SCORE, Schistosomiasis Consortium for Operational Research and Evaluation, ZAMREC, Zanzibar Medical Research Ethics Committee, ZEST, Zanzibar Elimination of Schistosomiasis Transmission, Behavior change, Elimination, Madrassa, Schistosomiasis, *Schistosoma haematobium*, Zanzibar

## Abstract

In Zanzibar, United Republic of Tanzania, Madrassa schools are influential institutions, where children and adults can learn about the interpretation of the Koran. We aimed to explore the involvement of Madrassa teachers for behavior change interventions in a randomized operational research trial designed to investigate the impact of multiple approaches to eliminate urogenital schistosomiasis transmission from Zanzibar. Madrassa teachers performing in the 30 communities of the behavior change study arm were trained in new interactive and participatory teaching methods by the local behavioral team and provided with schistosomiasis-teaching tools for teaching about transmission and prevention in their Madrassa. In July 2014, in a qualitative research study, we conducted 25 semi-structured interviews with Madrassa teachers to find out how they perceived their involvement in interventions against schistosomiasis. In 2014, 5926 among the 8497 registered Madrassa students in 30 communities on Unguja and Pemba islands received health education and participated in interactive behavior change exercises about schistosomiasis. Madrassa teachers reported that they valued their inclusion in the study and the opportunity to educate their students about schistosomiasis transmission, prevention, and treatment. They also perceived personal and community benefits as a result of their training and strongly supported the inclusion of additional Madrassa teachers in future intervention activities. Madrassa teachers are influential in the Zanzibari society, and hence are important change agents within our community-level behavioral intervention. They might constitute an untapped resource that can help to expand and increase acceptance of and participation in schistosomiasis and other neglected tropical disease control activities in African Muslim communities.

## Highlights

• Madrassa teachers participate in behavior intervention to eliminate schistosomiasis.
• Teachers valued the opportunity to educate students about schistosomiasis.
• Teachers can be influential and effective change agents in the intervention.
• Teachers can help to expand and increase acceptance of elimination activities.
• Teachers can help to increase participation in elimination activities.

## Graphical abstract

Madrassa teachers are influential in the society and hence important change agents within our community-level behavioral intervention for the elimination of urogenital schistosomiasis transmission in Zanzibar.

**Figure:**
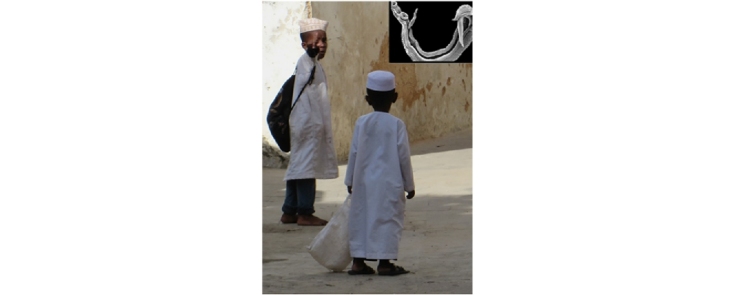

## 1 Introduction

Schistosomiasis imposes a significant health burden on people living in tropical and sub-tropical regions of Africa, Central and South America, and Asia and globally more than 230 million people are infected (Gryseels et al., 2006, Steinmann et al., 2006, Vos et al., 2012). On the Zanzibar islands belonging to the United Republic of Tanzania, urogenital schistosomiasis caused by *Schistosoma haematobium* has been highly prevalent in the past century (McCullough and Krafft, 1976, Savioli et al., 1989, Mgeni et al., 1990). However, rigorous schistosomiasis control interventions and likely also an increasing socio-economic standard and improved access to water and sanitation have resulted in a decrease of cases and an overall prevalence of below 10% in school-aged children (Stothard et al., 2009, Guidi et al., 2010, Knopp et al., 2013a, Knopp et al., 2013b). In 2011, the Zanzibar Elimination of Schistosomiasis Transmission (ZEST) alliance was formed with the aims to eliminate urogenital schistosomiasis as a public health problem from Pemba island and to interrupt transmission of urogenital schistosomiasis in Unguja island (Knopp et al., 2012). With the support of the Schistosomiasis Consortium for Operational Research and Evaluation (SCORE) a randomized operational research trial is being conducted in 45 administrative areas (shehias) on Unguja and Pemba, respectively, from 2011 until 2017, to assess the effectiveness of three schistosomiasis control interventions: (i) mass drug administration (MDA) with praziquantel; (ii) MDA and snail control; and (iii) MDA and behavior change activities (Knopp et al., 2012, Knopp et al., 2013a).

The behavior change component in arm iii is guided by the Human Centered Design (HCD) methodology, in which the researcher works in partnership with community members to design a solution to a specific problem through a participatory process. Through the HCD approach applied in Zanzibar in 2011, four major components were identified as important for the schistosomiasis behavior change intervention (Person et al., 2016b): (i) training schoolteachers, coaches, and other authorities in local public primary schools and religious schools in new interactive and participatory teaching methods in order to teach students about schistosomiasis; (ii) installing locally designed male and female urinals near contaminated natural open freshwater bodies where children congregate for use by children; (iii) providing safe play activities and play structures for children that are an alternative to playing in contaminated freshwater sources; and (iv) providing laundry washing platforms in areas located a short distance from local safe water sources to prevent children from washing clothes in contaminated freshwater bodies.

The HCD process also identified Madrassas, religious educational institutions, as important community venues, where children can be reached for health communication messages and behavior change activities. On the Zanzibar islands, more than 99% of the population is Muslim (US Department of State, 2014). As a result, religious education through a Madrassa is compulsory for most Zanzibari children and Madrassa teachers are influential in the community. Although the primary role of Madrassa teachers is Islamic education, teachers also engage students in a wide variety of topics including health and hygiene (Boyle, 2004, Blanchard, 2008). Hence, Madrassas and their teachers were included within the behavioral school intervention component. Madrassas are complimentary to government schools and many children in Zanzibar attend the two schools simultaneously. Hence, health education and behavior change activities provided within a Madrassa may overlap with similar activities provided in a government school.

The qualitative research study presented here is a process evaluation of a Madrassa teachers training, which was conducted in 2014 as part of the educational component for the behavior change interventions implemented in our randomized trial. This process evaluation study explored the knowledge, attitudes, and experiences of Madrassa teachers who have been trained in schistosomiasis prevention and control intervention activities and are currently participating in the SCORE behavioral study component in 15 shehias on Unguja and Pemba, respectively. Future studies will reveal the impact of the behavior change interventions on schistosomiasis transmission in Zanzibar.

## 2 Methods

### 2.1 Ethical considerations

This qualitative research inquiry was conducted in May–July 2014 as part of process evaluation activities approved by multiple institutional and ethical review boards for the overall operational research trial, and included ethical approval from the Zanzibar Medical Research Ethics Committee in Zanzibar, United Republic of Tanzania (reference no. ZAMREC 0003/Sept/011), the “Ethikkommission beider Basel” (EKBB) in Switzerland (reference no. 236/11), and the Institutional Review Board of the University of Georgia in Athens, Georgia, United States of America (project no. 2012-10138-0). Verbal informed consent was obtained from every Madrassa teacher participating in in-depth interviews.

### 2.2 Study setting

This qualitative study was conducted on Unguja and Pemba islands between May and July 2014. The Zanzibar archipelago is part of the United Republic of Tanzania and consists of the two main islands of Unguja and Pemba with an estimated resident population of 1.3 million (OCGS, 2013). Islam is the predominant religion and Kiswahili is the predominant language. Both Unguja and Pemba are divided into districts: six districts on Unguja and four districts on Pemba. On Unguja, urogenital schistosomiasis is endemic in all except the South district; on Pemba, all four districts are endemic for the disease. The districts are sub-divided into administrative units called shehias. Each shehia has one local, elected leader called a sheha, who is involved in political and administrative issues at the community level.

### 2.3 Training of madrassa teachers

**Fig. 1:**
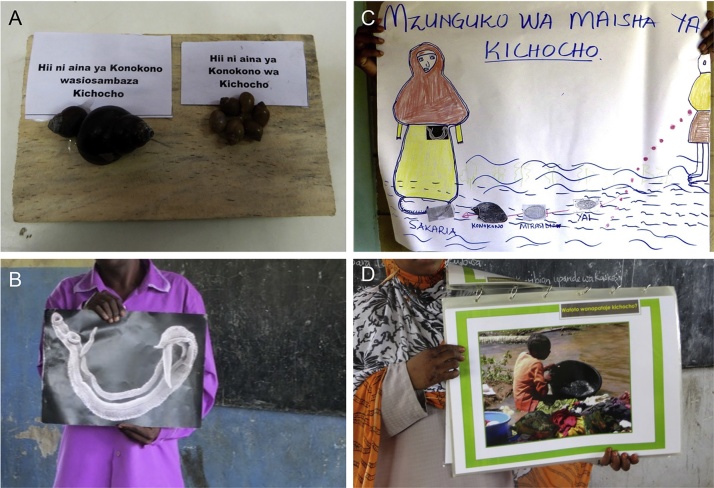
Teaching tools: (A) snail board (showing the intermediate snail host *Bulinus globosus* that transmits the parasite and one other snail species that does not support the parasite); (B) laminated picture of the blood flukes (male and female schistosomes) infecting humans; (C) picture of the life cycle drawn by the teachers themselves; (D) a flip chart (a community designed, interactive tool in Kiswahili that facilitates education on risk factors, transmission, treatment, and prevention of schistosomiasis).

The behavior change teams on Unguja and Pemba consist of four local research assistants who are responsible for the day-to-day activities within the behavioral intervention. In order to participate in the behavioral intervention, Madrassa teachers were expected to participate in a teacher training designed and co-taught by the senior behavioral scientist and co-investigator of the study. The seminar activities were meant to engage the teachers in interactive and participatory activities, instead of lecturing and rote memorization. Madrassa teachers from the 15 shehias belonging to the behavioral study arm on Unguja and Pemba, respectively, attended three, daylong interactive training sessions at the main office of each behavior change team. The teacher training sessions, led by the senior behavioral scientist and the behavioral teams, included topics on the life cycle, transmission, symptoms, and treatment of schistosomiasis. The teacher training sessions were designed according to the principles of Adult Learning Theory (Taylor and Hamdy, 2013). The sessions also included new participatory teaching methods, tools, materials, and games. At the end of the training, the teachers were provided with unique teaching tools designed in partnership with the community during the HCD process, to take back to their classrooms and to use in the education of their students. As shown in Fig. 1, teaching tools included a snail board (showing the intermediate snail host *Bulinus globosus* that transmits the parasite and one other snail species that does not support the parasite), a laminated picture of the blood flukes (male and female schistosomes) infecting humans, a picture of the life cycle drawn by the teachers themselves, and a flip chart (a community designed, interactive tool in Kiswahili that facilitates education on risk factors, transmission, treatment, and prevention of schistosomiasis). After receiving the training, teachers were requested to return to their classrooms and train their students about schistosomiasis. Teachers were asked to use the same interactive, hands-on teaching methods that were used during their training sessions.

### 2.4 Safe play

The HCD process also suggested a bi-annual ‘Kichocho Day’ event (Kichocho is the Kiswahili word for schistosomiasis), to encourage safe play behaviors and provide an alternative to playing at the river, as an important way to reach school-aged children rather than a typical didactic sensitization effort. Events at a Kichocho Day include safe games like tug-of-war, jump rope, netball, and soccer, as well as poems, songs, and dramas about schistosomiasis. Each activity has an educational component on how schistosomiasis can be transmitted and prevented. Kichocho Day events were initially planned for government primary schools that are also partners in the intervention. The Madrassa teachers, trained in safe play activities, requested their own Kichocho Day for Madrassas.

### 2.5 Study design and data collection

Using qualitative research methods, our aim was to understand Madrassa teachers’ experiences with the behavioral intervention trainings, subsequent implementation of behavioral activities post training, and their ideas for potential methods of scaling up the intervention through other Madrassas. Informed consent documents and an interview guide were developed in English and translated into Kiswahili (Corbin and Strauss, 2007, Kvale and Brinkmann, 2009). All forms and guides were pre-tested and modified to adapt to local linguistic and cultural nuances by the behavioral research team. All present Madrassa teachers, who had attended a schistosomiasis education and behavior change training conducted by the behavioral team and then proceeded to conduct education in their Madrassa classrooms, were recruited for in-depth interviews and included in the sampling frame (Strauss and Corbin, 1994, Patton, 2002, Patton, 2005, Corbin and Strauss, 2007). During the months of May–July 2014, semi-structured in-depth interviews were conducted in Kiswahili by an English and Kiswahili speaking research assistant, with some assistance from members of the local behavioral intervention team whose first language was Kiswahili. The interviews, approximately 25 min in length, took place inside of or nearby the Madrassa building. All interviews were recorded and later transcribed verbatim. Moreover, when agreeing to participate in the in-depth interviews, we gathered school demographic data from each Madrassa teacher.

### 2.6 Data management and analysis

Data were collected using an Olympus 70 audio recorder. Additional field notes were handwritten during the interviews and reviewed during debriefing sessions to verify accuracy of the interview session (Creswell, 1994). Narrative data were transcribed into English with review following translation to ensure accurate translation and local meanings.

We used a qualitative descriptive thematic analysis using a modified grounded theory coding approach. Hence, data analysis began with analysis of the first few teacher interviews allowing for emerging, unexpected, and/or inconsistent issues to be explored in subsequent interviews. The first author of this paper was the primary data coder (MC), guided by the senior social scientist (BP), with verification of interpretive codes by the local behavioral team research assistants. Open, axial, and selective coding was used to analyze the narratives (Miles and Huberman, 1994, Charmaz, 2006). A coding frame was developed through open coding, a word-by-word analysis, used to identify, name, and categorize explanations and descriptions of the day-to-day reality of Madrassa teachers as related to the schistosomiasis behavioral intervention (Miles and Huberman, 1994, Charmaz, 2006). Consensus on the coding frame was obtained through discussions with the local Zanzibari behavioral research assistants. Axial coding, the process of relating codes to each other, via a combination of inductive and deductive thinking, was used for analysis of specific emergent themes, across themes, and for the relationships between themes. The major themes that emerged were the positive experiences of the Madrassa teachers during the training, including the discussion of the training methods and materials; the benefits of the trainings to the Madrassa teachers themselves, the students, and the community as a whole; the experience of taking the training to the students; and the value of giving this training to all Madrassa teachers. Over the course of data collection, emergent themes became redundant, suggesting that all major themes had been identified and saturation obtained. An analysis matrix served as a framework for the resulting findings. The trustworthiness of our data was derived from standardization of methods and documentation for auditability, and verification of data findings with local staff members (Devers, 1999).

## 3 Results

### 3.1 Participating Madrassa teachers

Thirty teachers participated in the behavioral training, but two were not present on Zanzibar during the time of the interviews. Three teachers from Pemba had recently replaced former teachers and had not yet received training or teaching tools. Hence, a total of 25 Madrassa teachers were included in the analysis. Among them, 14 were from Unguja and 11 from Pemba. The average age of participants was 43 years and the age range was 22–75 years. On average, participants had been teaching in a Madrassa for 13.3 years. The narrative quotes from Madrassa teachers noted below represent responses from unique interviewees.

### 3.2 Madrassa students

According to the school records reviewed by the behavioral teams, students attending Madrassa ranged from three to 65 years of age. The average reported age across all schools for the youngest student attending Madrassa was five years and for the oldest student attending Madrassa was 32 years. Nine Madrassa teachers reported that adult learners study at their Madrassa. On average, teachers hold 2.3 classes per day. The teachers on Pemba reported having an average class size of 212 students, while the teachers on Unguja reported having an average class size of 184 students. Process data collected by the behavioral teams on standardized reporting forms estimated that 5926 among 8497 Madrassa students received health education and participated in interactive behavior change exercises about schistosomiasis in the 15 study shehias on Unguja and Pemba, respectively. New students who had entered school around the time of the data collection had not yet received the intervention.

### 3.3 Madrassa teachers’ experiences with training sessions

Madrassa teachers reported that they were very satisfied with the annual schistosomiasis training sessions and the new teaching methods learned. They reported that the participatory teaching methods were more practical, interactive, and understandable and that the trainings provided a comprehensive understanding of disease transmission, symptoms, treatment, control, and prevention. Madrassa teachers reported an increase in self-efficacy or confidence about their ability to teach about schistosomiasis topics. The new teaching methods, teaching tools, and materials engaged teachers and taught them to engage students more effectively instead of limiting their interactions to lecturing and rote memorization. A Madrassa teacher involved in a training on the prevention of another disease expressed a preference for the interactive nature of the schistosomiasis training,

*“The [other] training was just lecture, there were no actions [participation]. Another difference is the kichocho training has teaching tools but the [other] program did not have. It is best to be educated through actions [participatory activities] because then you will not forget. You will remember more than through lectures/discussion. Actions [participatory activities] are better than lecture.”* (32 year-old male, Pemba).

Another Madrassa teacher talked about the content of the training related to disease transmission and the life cycle drawing each teacher makes,

*“We were taught what is kichocho. The blood worm that is causing kichocho, the effects of kichocho, and the way it affects the human body. We were shown the way it spreads. It begins in the person and goes in the river during urination. The child releases the eggs [in the urine] and the parasite looks for a snail. He is changing and becoming a different type [after the snail] and when he meets with a person he enters [the person]. Then they taught us the way we should protect ourselves against kichocho. We should educate our students about avoiding bathing and playing in the river.”* (42 year-old male, Unguja).

The behavioral teams’ yearly seminars, along with their ongoing relationship with the religious teachers were reported as a valued experience. A teacher said,

*“I like that we are continuing to be educated. Every few weeks the kichocho team is passing through to educate us some more*.” (28 year-old male, Unguja).

### 3.4 Madrassa teachers’ perceived benefits from the training sessions

Teachers identified personal and community benefits as a result of attending the teacher training. Madrassa teachers reported a limited knowledge of schistosomiasis before beginning to work in our project. As a result of the training, teachers reported a significant increase in their own knowledge about schistosomiasis transmission and the parasite life cycle, risk factors for children and adults, disease symptoms, treatment, and prevention, as well as how to dispel local myths about the disease. A Madrassa teacher from Unguja said,

“*I did not know how kichocho is transmitted. Before the training, I did not know about the appearance of the kichocho worm. I use to hear that kichocho is transmitted from the water but I had no idea on how kichocho got in to the body. Now I know that in the river or ponds there is a kichocho snail and that there is a kichocho worm and the kichocho eggs are passed when a person with kichocho pees in the water. I now know that from the trainings.”* (46 year-old male, Unguja).

Madrassa teachers reported new skills and behavioral capabilities in using teaching aids and teaching tools, particularly around transmission of disease and the importance of sharing that with community members. Another Madrassa teacher from Unguja reported,

*“We have gained this skill in kichocho education and it has become like a calling to us. It is now like our responsibility in the society. The behavior change is not only for us as educators but to others. Preventing people not to get kichocho and making sure that they don*’*t play in infected water has become our job. We are now taking all precautions to prevent kichocho when passing near the water. It is not like before when we were not worried at all about it.”* (53 year-old male, Unguja).

Several Madrassa teachers reported that they had previously never taken the drugs during MDA and now they do. A teacher reported on his personal behavior change,

*“Before I got training, I was given four kichocho tablets [at the MDA], I divided them in two. But after getting the training, when I was given four tablets I ate all. I now know the importance of the medicine. I educate other people about the use of kichocho medicine. Because many of us, we are given three or four tablets and we are only eating half. If you only eat half, the kichocho parasite doesn*’*t die. He is ‘intoxicated’ but then he is continuing like normal and continuing to attack [the infected person]. But if you swallow all the tablets he dies.”* (28 year-old male, Pemba).

Madrassa teachers overwhelmingly reported that they perceived their training as a benefit to their students and their community. Due to the significant amount of time that Madrassa teachers spend with their students, and their social standing in the community, the teachers identified themselves as major change agents for the behavior change intervention. One man reported,

*“We are the caretakers of the children and the children are the ones causing the problem, so it is very important that Madrassa teachers have expertise in protecting ourselves against the problem because in society, Madrassa teachers are the caretakers of society. So because he is the caretaker of society it is necessary he protects against problems that are coming into society, and one of the problems is this disease of Kichocho.”* (50 year-old male, Pemba).

### 3.5 Implementation of training and student reaction

Teachers reported that students reacted positively to their schistosomiasis trainings. All the teachers reported that they were using the teaching tools to educate their students.

*“In educating the students I put them in groups and then I used the teaching tools we were given, like the snail board, blood fluke picture, blood vessel picture, the life cycle picture that we drew, all of the tools I used to teach the children.”* (45 year-old female, Unguja).

Madrassa teachers reported that students were interested in all of the classroom teaching tools but were most surprised by the blood fluke. A major focus of our intervention was shifting the perception of the blood fluke from a simple “worm” that everyone gets, to a dangerous parasite that can lead to serious health consequences if untreated. The Madrassa teachers consistently reported the blood fluke picture as the most important teaching tool for motivating children to take the medicine for schistosomiasis and staying out of contaminated freshwater. A teacher reporting on children’s reactions to the blood fluke teaching tools said,

*“[After seeing the blood fluke] the students were very startled. They didn*’*t know about it. They thought it was just a normal worm. But it is not a normal worm. The kichocho worm is very different from the other worm. So they were very surprised*.*”* (28 year-old male, Unguja).

One Madrassa teacher reported that he conducted education sessions at the river, and took his students directly to the river for real-world instruction. While there, he also provided education about schistosomiasis transmission to children who were washing or playing in the contaminated water at the time.

In addition to classroom teaching tools, Madrassa teachers were taught about risky behaviors associated with water play and introduced to new outdoor safe-play activities and approaches. Ten Madrassa teachers had attended at least one *Kichocho Safe Play* event at a government school with many bringing their students to the event as well. A Madrassa teacher talked about safe games,

*“I talk to students as well as show them the blood fluke picture I was given from the training. Then they play the safe games. The games they play at Madrassa they are also playing these games at home now. The ones they learn at school. They also sing kichocho song at home. This has helped to increase knowledge because everyone is talking about kichocho.” (57 year-old male, Pemba).*

Teachers also reported discussing with students and parents that praziquantel was the only treatment that kills the schistosome parasite, the necessity of taking praziquantel tablets on a full stomach, and the importance of swallowing all praziquantel tablets at the same time. Madrassa teachers promoted the important behavior change message to swallow all the tablets the day they are given,

*“I told them the importance of the medicine. There are some children who toss away their medicine. I told them there is no tossing the medicine away. I educated them well. I told them the importance of the medicine, and when you swallow it, it will kill the parasite that is causing kichocho. The students listened, and they swallowed the medicine.”* (23 year-old male, Unguja).

Overall, Madrassa teachers expressed positive experiences during the training sessions, described positive personal and community benefits derived from the trainings, and reported using the new knowledge and skills with students and community members for the prevention, control, and treatment of schistosomiasis. The Madrassa teachers expressed a need for including more Madrassa teachers as a means of increasing the reach of the behavioral intervention. The teachers involved in the current intervention represent a very small portion of the total Madrassa teachers across the 15 study shehias in Unguja and Pemba, respectively. They also expressed a need for more resources to continue their activities after the trial is completed.

## 4 Discussion

Similar to other health intervention studies including religious leaders (Prihartono et al., 1994, Mfaume et al., 1997, Kanoa et al., 2006, Hipple and Duff, 2010, Pach et al., 2013, Sarma and Oliveras, 2013), the emergent data from our study suggested that following behavior change trainings, Madrassa teachers perceived themselves as influential change agents and trusted sources of information for their students and other people in the community in the fight against schistosomiasis.

Madrassa teachers considered themselves as important for reaching children too young to attend public schools, or those whose families cannot afford to send them to school. This has important implications because children not attending school may have greater exposure to the parasite through water associated domestic chores such as washing clothes and gathering water for household uses. These children may also spend more time playing in open natural freshwater, which can lead to further transmission of schistosomiasis.

The actual theory-based education and behavior change training sessions, with new teaching methods and materials, were well received by the Madrassa teachers. The training sessions were the same trainings conducted with schoolteachers in the government study schools but tailored to the learning needs of Madrassa teachers. The Madrassa teachers appraised these trainings as valuable and equitable learning opportunities that increased their status within the community because they were similar to the teacher trainings. The new teaching methods engaged teachers and taught them to engage students in their own learning activities. The training sessions drew upon teachers’ own life experiences and knowledge as leaders in the study communities, addressed a perceived need among themselves and their students, and treated participants with respect (Collins, 2004, Taylor and Hamdy, 2013).

For most Madrassa teachers this was the first time they had been asked to participate in a health initiative. An emphasis was placed on the importance of the Madrassa teachers’ leadership role in the community and their vital responsibility in communicating the behavior change messages. Teachers reported that they enjoyed the training and valued their inclusion in the study. Many cited the participatory and interactive approach of adult learning theory as a novel, positive feature of the training sessions that they carried over as a teaching strategy with students in other topics. They also valued the consistent interaction they have with the behavioral research team throughout the study. A striking finding was that prior to the training few Madrassa teachers had taken the drug praziquantel against schistosomiasis and after the training all Madrassa teachers reported taking the drug during MDA efforts unless not in the village at the time of distribution. The Madrassa teachers also reported that they perceived themselves as role models and caretakers for the community members to take the drug correctly in order to eliminate schistosomiasis in Zanzibar.

The Madrassa students were reported to enjoy and actively learn about schistosomiasis in a manner similar to the teachers. Our previous work in Zanzibar revealed that children engaged in high risk behavior for acquiring schistosomiasis (Person et al., 2016a) and that students were concrete thinkers and wanted and needed real-world examples and information. All the teaching tools and aids were visually oriented and tangible examples: the snail board, the blood fluke picture, the flip chart, and the life cycle picture. These concrete tools allowed students to see real world examples and Madrassa teachers to answer questions in the same manner. The students were reported to enjoy the training and were particularly startled by the picture of the blood fluke. This is an important finding as one objective of the training was to convey the danger of the blood fluke and to emphasize that it is not merely an intestinal parasite.

There is a growing recognition for the need to supplement preventive chemotherapy with improved access to water and sanitation, snail control and behavior change interventions in schistosomiasis elimination campaigns (Utzinger et al., 2009, Rollinson et al., 2013, WHO, 2013). It is necessary to assess the effectiveness of these interventions in order to provide an evidence-base for other countries that aim to eliminate schistosomiasis (Gray et al., 2010, Rollinson et al., 2013). Madrassa teachers occupy an influential position within Islamic communities. Due to their responsibilities as educators and community leaders, they come into contact with hundreds of students and community members each week. Because of their importance in the community and their sphere of influence, trained Madrassa teachers can serve as effective health educators and behavior change agents within health interventions and they have the potential to change knowledge and practices related to the transmission of multiple diseases, including schistosomiasis.

Qualitative research, such as the research described here, can provide valuable insight into the implementation of behavioral interventions, for neglected tropical disease prevention and control in developing countries and can inform policy and decision-making to enhance community member health (Chandler et al., 2008, Person et al., 2008, Jefferds et al., 2010, Bauch et al., 2013). Sharing the voices of community members actually implementing novel behavioral interventions can provide credibility and insight to other community members faced with similar public health challenges.

However, while this study sheds light on the current role of Madrassa teachers in the behavioral intervention of a randomized operational research trial in Zanzibar there are important limitations. Data were collected in Kiswahili, while analysis was conducted in English. Important nuances may have been missed or ideas misinterpreted. A single individual conducted the data coding with only review by a secondary person. Data interpretation while reviewed by local staff may have biases not immediately apparent. While this inquiry was conducted across teachers on two different islands, Unguja and Pemba, the results are not generalizable to all Madrassa teachers in Zanzibar and elsewhere.

## 5 Conclusions

We believe that Madrassa teachers are an important and untapped resource for health communication in communities across Zanzibar. The teachers have the potential to serve as important change-makers in our urogenital schistosomiasis elimination program. Other schistosomiasis and neglected tropical disease programs throughout Africa could enhance the reach and credibility of their community level, behavioral interventions by including local religious leaders as change agents.

## Potential conflicts of interest

All authors: no conflicts.
